# Hyaluronic acid fillers for cosmetic and functional correction in thyroid eye disease

**DOI:** 10.1111/jocd.16541

**Published:** 2024-08-20

**Authors:** Ilaria Proietti, Francesca Svara, Chiara Battilotti, Agnieszka Dybala, Tolino Ersilia, Nicoletta Bernardini, Nevena Skroza, Concetta Potenza

**Affiliations:** ^1^ Dermatology Unit “Daniele Innocenzi” “A. Fiorini” Hospital, Terracina (LT), Sapienza University Polo Pontino Italy

To the Editor,

Thyroid Eye Disease (TED), also known as Grave's Ophthalmopathy, is an autoimmune disorder characterized by inflammation of orbital and periorbital tissues, leading to ocular symptoms like proptosis, eyelid retraction with corneal exposure and diplopia. About 50% of Graves' disease patients develop TED, with increased risk in women, smokers, and those undergoing radioactive iodine therapy.^1^ TED usually emerges concurrently with hyperthyroidism, with an initial inflammatory phase lasting up to 2 years followed by a quiescent phase, during which symptoms may plateau or improve. While mild TED is usually self‐limiting, moderate‐to‐severe cases often persist, with proptosis being the least likely to resolve. Treatment varies based on disease activity and severity, emphasizing achieving euthyroidism and smoking cessation. Mild cases may require symptomatic relief and selenium supplements, while moderate‐to‐severe active TED may necessitate glucocorticoids, orbital radiotherapy, or immunomodulatory agents. In stable phases, surgical interventions may restore function and aesthetics.^2^ Minimally invasive options such as Hyaluronic acid (HA) and Botulinum toxin type A (BTX‐A) offer alternatives for ineligible or surgery‐averse patients, providing fine‐tuning postoperatively.

## CASE PRESENTATION

**FIGURE 1:**
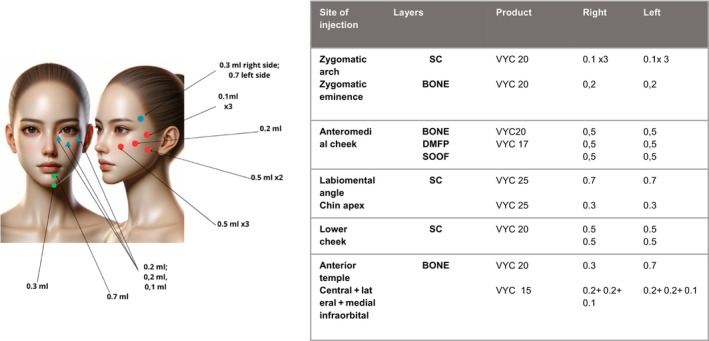
Treatment plan with Vycross hyaluronic acid (HA).

**FIGURE 2:**
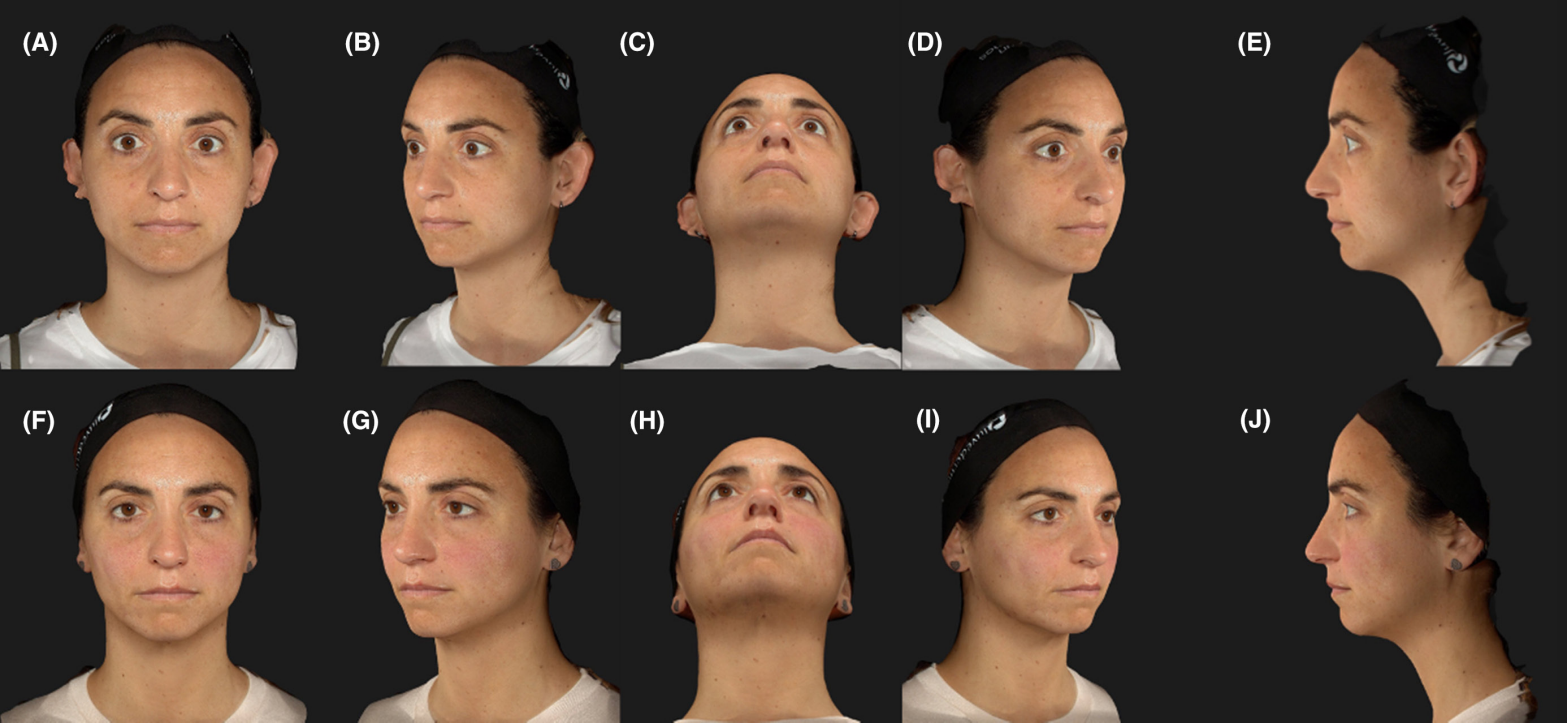
Frontal, left lateral 37°, underside, right lateral 37° and left side views at baseline (A–E); frontal, left lateral 37°, underside, right lateral 37° and left side views after 12 months from the Vycross HA treatment (F–J).

We present a case of a 42‐year‐old woman who was referred to our dermatology ambulatory with a history of bilateral TED. The patient reported a diagnosis of Graves' disease at the age of 38, associated with significant ocular symptoms. She underwent antithyroid therapy for 18 months, experiencing an improvement, but the ophthalmic condition persisted. Upon clinical evaluation, the patient reported weight loss in the past few months, accompanied by tachycardia. Significant eyelid retraction and proptosis were observed with aesthetic and functional discomfort, indeed the patient reported dryness and irritation. We decided to start treatment utilizing Vycross® HA filler. All injections were performed bilaterally, and the procedure was repeated over three separate sessions, spaced 1 month apart, for a total treatment period of 2 months. The following injection sites and volumes were used: three needle injections of 0.1 mL each were administered into the zygomatic arch (malar region). An additional injection of 0.2 mL was placed into the mid‐cheek area at the bone level. In the lower cheek, one needle injection of 0.5 mL was performed at the bone level, followed by two more injections of 0.5 mL each using a 22‐gauge cannula (70 mm length) at the deep muscular facial plane (DMFP) and the suborbicularis oculi fat (SOOF) levels, respectively. Two injections of 0.5 mL each, using a 22‐gauge cannula (70 mm length), were conducted at the subcutaneous level in the lower cheek region. Two subcutaneous needle injections of 0.7 mL and 0.3 mL were administered in the labiomental angle and chin apex areas, respectively. Finally, a 0.3 mL needle injection at the bone level of the right temple, and a 0.7 mL injection on the left temple were administered, followed by one needle injection of 0.2 mL in central and lateral infraorbital area and 0.1 mL in the medial infraorbital area. There were no reported adverse effects and the result was extremely pleasing and remained stable at the 6 and 12‐month follow‐up visit (Figures 1 and 2).

## DISCUSSION

Managing TED is challenging. Surgical options, such as orbital decompression, strabismus correction, and eyelid surgery carry risks and uncertain outcomes, especially in acute cases, as well as prolonged recovery.^2^ Recently, noninvasive technique like HA fillers and BTX‐A are emerging. BTX‐A injections can be associated with ptosis and ecchymosis, requiring careful administration and monitoring.^3^ Additionally, its effects have a relatively short duration, necessitating repeated administrations.^4^ In contrast, HA fillers addresses both aesthetic and functional challenges with minimal invasiveness and reversibility thanks to the possible use of hyaluronidase and adjustability through repeat treatments.^5^ Vycross® fillers provide durable volume and structural support for correcting proptosis and eyelid retraction, with reduced inflammation and side effects compared to other fillers, ensuring natural results and better tissue integration.^6^ The patient was in the stable phase of TED, which is ideal for interventions as inflammation had subsided. Specific injection sites were carefully chosen to restore facial symmetry and volume, particularly in the cheek and temple areas, commonly affected in TED patients. Precise targeting across various anatomical planes offered a more balanced correction compared to singular injections. A combination of cannulas and needles was chosen to reduce the risk of bruising and swelling and ensure even filler distribution. This technique is particularly important for TED patients, who may have sensitive and inflamed tissues. The treatment involved three sessions spaced a month apart to allow gradual improvement and manage complications, with carefully calculated volumes at each site to ensure balanced, natural results and avoid overcorrection.

## CONCLUSION

Our study underscores the efficacy and safety of nonsurgical approaches such as HA fillers, as a versatile alternative for patients ineligible or averse to surgical interventions. Aesthetic interventions must consider the disease's activity and timing, ensuring treatments are administered after the inflammation has subsided to prevent complications like granulomas or nodules. In this particular case, we employed Vycross® technology to demonstrate promising outcomes, stability and favorable results over a 12‐month follow‐up. However, to make definitive claims about the safety, effectiveness, and favorable outcomes of HA fillers in the treatment of TED, larger and more comprehensive studies need to be conducted. Future research with a larger sample size will be necessary to confirm these preliminary findings and to establish standardized treatment protocols.

## AUTHOR CONTRIBUTIONS

All authors were responsible for the concept and design of the study, collection and collation of data, analysis, and interpretation of data, write an article, reviewing this article, final reviewing this article and graphics performance.

## CONFLICT OF INTEREST STATEMENT

All authors declare that they have no conflicts of interest.

## ETHICS STATEMENT

This study was reviewed by local institutional review board (Ethics Committee of the Medical University Sapienza: Approval number US2345). Written consent is given by patient.

## Data Availability

The data that support the findings of this study are available from the corresponding author upon reasonable request.
